# Dr. Magennis, on the Cure of Dropsy

**Published:** 1800-09

**Authors:** James Magennis

**Affiliations:** Physician to the Royal Hospital, Plymouth


### *To the Editors of the Medical and Phyfical Journal.*

Gentlemen,

YOUR valuable and truly ufeful Journal needs no encomiums of mine to point out its various merits to an enlightened and difcerning public; its extenfive circulation through the Britifh dominions, and the great refpectability of many of your Correfpondents, among whom I am happy to notice the name of Dr. Denman, are the fureft pledges of its ftability and increafing celebrity.

The readinefs with which you communicate to the public all facts and obfervations that have a tendency to enlarge the boundaries of Medical Science, or alleviate the miferies of human nature, induces me to requeft the infertion of the following cafes of fuccefsful treatment of Dropfy, in your next monthly Mifcellany.

In the plan of treatment and cure adopted in thefe cafes, I have have not to boaſt the diſcovery of any new remedy, either chemical or botanical; no new ſpecific to propoſe, or arcanum to lay open; in this reſpect, the lovers of novelty and wonder will certainly be diſappointed: But although the medicines employed have been long known and eſteemed almoſt by every Tyro, as well as by men of the firſt ability in the profeſſion, yet it muſt be admitted, that when theſe are united, and variouſly combined with no properties chemically repugnant, they may poſſeſs energies and active powers, which ſeparately and diſtinctly adminiſtered, they would wholly fail in.

In the cure of every ſpecies of Dropſy, practitioners in general have two indications in view, that of evacuating the ſuperabundant fluid in the firſt inſtance; and, ſecondly, to guard againſt its farther accumulation. To accompliſh theſe deſirable ends, recourſe is almoſt invariably had to the operation of draſtic purges, frequently exhibited and repeated, ſuch as ſeneka, jalap and calomel, the neutral ſalts, and the occaſional uſe of diuretics. When by a due perſeverance in theſe means, the water is totally or in great meaſure expelled, different tonics, as bark, ſteel, and bitters are employed to reſtore the loſt tone of the conſtitution, and confirm the cure. But although this treatment may have frequently ſucceeded in removing the diſorder in queſtion, yet there is great reaſon to believe that it has been only in young and naturally robuſt habits, where the complaint was of recent ſtanding, and not depending on any previouſly exiſting diſtemper. For, if we are to conſider Dropſy as a diſeaſe commonly of high debility, and conſequently of diminiſhed excitement, whether originally produced by profuſe evacuations of blood, ſuddenly inducing a conſiderable diminution of the vital powers, or by long protracted acute diſeaſes, or other cauſes occaſioning general atony and relaxation of the fibres, we ſhall naturally and juſtly be led to conclude, that theſe violent evacuant remedies have rather a tendency to increaſe the malady by ſtill farther exhauſting the excitability or ſenſorial power, than to remove it.

That this practice is often attended with great danger, and ſometimes with fatal conſequences, we have the teſtimony of Dr. Milman, who ſays, that after the Dropſy is removed, the patients will ſometimes die without any evident cauſe; and again, this reſpectable author obſerves, that we muſt take care not to make too free with the uſe of purgatives if the patient be weak. I might aſk the Doctor with much propriety, if he had ever met an inſtance of dropſy, aſcites, or anaſarca, as a general diſeaſe, that was not accompanied with and preceded by great debility? Notwithſtanding theſe cautions, and their manifeſt propriety, the Doctor however depends, as Sydenham

 and and others did before him, chiefly on the good effects of purgatives; for he speaks, among other things, of the advantages arising from a decoction of seneka procuring nine or ten stools a day. Sydenham advises cathartics to be administered every day, unless through the too great weakness of the body, or the violent operation of the purgative, it shall be necessary to interpose a day or two now and then; because, he says, if considerable intervals be allowed to occur between the exhibition of the purgatives, the water will be afforded an opportunity of again collecting.

A careful and diligent investigation into all the phenomena attending Dropsy, will convince every impartial and unbiassed observer, that it is always a disease of diminished, and never of increased excitement; unless when excitement is pushed beyond its due bounds into indirect debility, as is frequently the case with men who indulge too freely in the luxuries and conviviality of the table, whose dissolution is not unusually preceded by this very disease: But still debility, whether of the direct or indirect species, and however induced, must precede and accompany it throughout all its stages.

If this view of the question be just, and founded on accurate observations deduced from the phenomena known invariably to attend the disease, how many lives, then, must annually have fallen a sacrifice to the common but mistaken practice? a practice grounded on false and erroneous theory; a practice which, instead of opposing the destructive tendency of the disease, is in direct unison with it; an accredited error sanctioned by ages, and maintained on the authority of great names, but which will be discarded the moment we have courage to reason from effects to causes, and to consider real facts divested of the influence of splendid but illusive systems. I am unacquainted with any cause which has operated more powerfully against the extension and improvement of medical science, than implicit confidence in certain illustrious names and systems of physic: The mind resting with a kind of fatal security on the supposed truths of those oracles, suspends its own inquisitive propensities and energies, thereby effectually shutting up all avenues to enquiry and investigation.

Convinced by experience of the inefficacy and pernicious consequences of employing evacuant and debilitating remedies for the cure of a disease depending on atony and relaxation, more especially of the vascular system, I pursued a course diametrically opposite.—I endeavoured to support and invigorate my patients by the most powerful and permanent stimuli, both with respect to medicines and diet; and with these were joined active diuretics, in order to stimulate the kidneys and determine mine the ferous fluid to thofe paffages. In fhort, to ftrengthen the habit, to reftore the energy and loft tone of the vafcular fyftem, to promote abforption, and finally to expel the ferous collections from the body, were the ends propofed: how far I fucceeded will be beft feen by a narrative of the following cafes.

### CASE I.

It is to be recollected that I began the treatment of this cafe on the old plan. James Griebel, a French prifoner, a young man 28 years of age, was admitted into the Hofpital at Norman Crofs on the 3d of November 1799; he dated the commencement of his complaint about three weeks before, occafioned probably by poverty and want. On examination, I found the abdomen very much enlarged with evident fluctuation, the legs much fwelled and œdematous, eafily receiving and retaining a long time every impreffion; a quick, fmall pulfe, a dry tongue, parched fkin, great thirft; paffed little or fcarcely any urine; a fhort troublefome cough; difficult refpiration, fo much fo that he was obliged to keep in a half erect pofture whenever he flept; a fallow, emaciated countenance, with large blotches and fcabby eruptions all over the body. He was immediately ordered a brifk cathartic of jalap and calomel, which procured feveral copious ftools and relieved his breathing. On the 5th the fymptoms became equally oppreffive and diftreffing. Rep. cathar. with fimilar effects, though lefs permanent. 6th, All the fymptoms recurred with additional violence, the patient being confiderably reduced. Ordered this day a diuretic mixture, Aq. menth. kali, acet. et tin. fcillæ. Paffed no water except when at the water-clofet, and then even very fcantily. 8th, Several watery evacuations by the anus yefterday; notwithftanding which, the legs, thighs, and abdomen were more enlarged than ever, with extreme debility and emaciation. It now became evident that the patient would fpeedily fink, unlefs relieved by other means. Under this idea he began the following mixture this day: ℞. Aq. menth. ℥vj. ferr. vitriol. kali. myrrh an. ℈j. tin. fcillæ. fpt. æth. nit. a. ʒifs. tinct. opii. gt. xl. fumat cochl. iij. 4is. horis. At the fame time he was ordered a pint of red wine, and nourifhing diet in fmall quantities frequently repeated. 9th and 10th, no alteration. On the 11th he made confiderably more water, but he was ftill greatly debilitated. 12th and 13th, the evacuations by the kidneys much increafed, fpirits better, ftrength amended. 15th, much better. The power of the mixture augmented: Ferr. vitr. kali. myrrh a. ℈ij. tinct. op. gt. l. tin. fcillæ. fpt. æth. n. a. ʒiij. aq. menth. ℥vj. ut antea. S. 17, Quantity of urine now daily 5 pints. 18th, Increafed to 6 pints, of a dufky brown colour with much fedi-

 ment; ment; treatment the ſame; all the bad ſymptoms greatly abated; the legs, thighs, and abdomen conſiderably reduced; reſpiration much relieved, thirſt gone, appetite good; in ſhort, he was wonderfully amended. 23d, Almoſt free from complaint, paſſing daily 6 pints and upwards of urine. His medicines and treatment continued the ſame till the 30th, at which time he was perfectly reſtored to health; and on the 4th of December he was diſcharged to priſon, having paſſed during the laſt 15 days near 90 pints of water, beſides what was loſt during operations by the anus. It is worthy of remark, that the tone and vigour of the fibres of the inteſtinal canal were by this time ſo completely reſtored, that he was obliged to take aperients twice, on the 24th and 29th. The ſcabby eruptions had at firſt much the appearance of being a ſpecies of itch; but before the 30th the ſkin was wholly cleared from them.

### CASE II.

John Colquhoun, aged 42 years, a ſeaman belonging to his Majeſty's ſhip Le Pompée, was admitted into the Royal Hoſpital at Plymouth on the 4th of June laſt, labouring under univerſal anaſarca; his legs and thighs were ſo aſtoniſhingly diſtended, that it was with the utmoſt difficulty he could ſtand; great proſtration of ſtrength, dejection of ſpirits, a troubleſome cough with expectoration, thirſt, difficult reſpiration, pain about the ſcrob. cordis, loſs of appetite, profuſe diarrhœa; paſſes no urine except at ſtool, and then only a few drops at a time. The hiſtory of the caſe previous to admiſſion: He had been affected with cough at leaſt twelve months; the ſwellings of the extremities began about five weeks before I ſaw him, and the diarrhœa was chiefly occaſioned by the purgatives employed to remove the dropſy; he was, upon the whole, much emaciated and reduced. The ſtate of his bowels became the firſt object of my care; and having by the uſe of the cretaceous mixture, tinct. catechu and opium, almoſt removed this complaint, he began the following mixture on the 7th: ℞. Ferr. vitriol. grs. xv. kali et myrrh a. ℈j. aq. menth. ℥vj. tinct. ſcillæ. ſpt. æther nitr. a. ʒjſs. tinct. opii gts. lx. M. capt. cochl. iij. 4is horis. This treatment was continued till the 11th, with little amendment. ℞. Aq. menth. ℥vj. ferr. vitr. ʒſs. tinct. ſcil. ſpt. æth. n. a. ʒijſs. tin. opii. gt. lxx. M. ut antea ſumend. wine, nouriſhing diet and drink without reſtriction. 13th, The diſcharge of urine conſiderable. 20th, The quantity amounted to about 5 pints, and continued to increaſe daily. 26th, The ſtrength of the mixture ſtill farther augmented to, Ferr. vitriol ℈ij. kali et myrrh a. ℈ijſs. tinct. ſcill. ſpt. æth. n. a. ʒiij. tinct. opii gt. lxx. aq. menth. ℥vj. M. From this time time the difcharge by the kidneys increafed daily, till it amounted to 6 pints and upwards of high coloured water, emitting a ftrong fœtor, and depofiting a heavy fediment. On July 10, his extremities were reduced to their natural fize, the cough and expectoration almoft wholly removed, the pain about the fcrob. cord. much abated, and his ftrength and appetite greatly amended. On the 11th, he had feveral copious motions; diarrhœa being extremely prevalent among the patients for fome time paft, which compelled me to fufpend the Chalybeate mixture, and to have recourfe to the cretaceous as at firft. In four days the diarrhœa was removed; and, on the 15th, he commenced the Chalybeate mixture, although every veftige of Dropfy was at this time wholly removed; but the diarrhœa had again reduced him. It is remarkable, that during the 13th, 14th, and 15th, he fcarcely made a quart of water each day.

This man was accidentally feen on board the Pompée, and ordered to the Hofpital by Dr. Harnefs, Commiffioner of fick and wounded Seamen; a gentleman, the fuavity and politenefs of whofe manners are only equalled by his humanity and profeffional abilities. From the marked appearance of hectic, joined to the anafarca, diarrhœa, and other bad fymptoms attending this patient, the Doctor confidered it a loft cafe.

### CASE III.

Henry Roach, 26 years of age, a feaman belonging to his Majefty's fhip Windfor Caftle, was received into the Royal Hofpital on the 1ft of June, 1800. I found the abdomen enormoufly enlarged, fluctuation, dry tongue, unquenchable thirft, the lower extremities much tumified and œdematous, an unceafing cough, and he expectorated largely a purulent kind of mucus, if I may be permitted the expreffion, flightly tinged or ftreaked with blood; a fixed and diftreffing pain about the fcorbicul. cordis, attended with profufe perfpirations daily towards morning! The abdomen was fo diftended, and his ftrength fo exhaufted, that he could neither ftand on his legs from the fize and weight of his body, or lie down for fear of fuffocation. He dated the commencement of his diforder from having put on a damp fhirt the 24th of February laft; from that time he had conftant cough, which gradually increafed till his belly began to fwell, about five weeks before his admiffion. Whatever cathartics could do had been already liberally tried on board fhip. His countenance had all the appearance of a completely formed hectic; the alæ of the nofe were pinched in, the cheeks funk and hollow, with a circular rouge-like tinge in the centre of each; in fhort, his whole appearance indicated approaching approaching diſſolution, or, at beſt, incurable phthiſis; nor had I the ſmalleſt hope of his recovery. For the firſt three days I preſcribed only a pectoral mixture with tinct. opii camp. to eaſe the violence of the cough, expecting that he could not ſurvive many days. On the 4th of June, however, he began with the Chalybeate diuretic mixture, which was continued till the 8th without much variation; but he made on this day near a quart of water at different times. Increaſed the ferr. vitri. to gr. xxv. kali. myrrh a. ℨſs. tinc. ſcillæ. ſpt. æth. nit. a. ℨiiſs. tinc. op. gt. xl. aq. menth. ℥vj. m. ſum. cochlear iij. 3is horis. Allowed a pint of red wine; no reſtrictions reſpecting the quantity of common drink, which was made by boiling ℨij. of crem. tart. for a few minutes in a quart of water with a little orange-peel, and ſweetened with honey. From this time to the 26th there was no alteration in the treatment, but the diſcharge by the kidneys increaſed to the aſtoniſhing quantity of between 8 and 9 pints of water daily, very high coloured, and depoſiting a copious ſediment; he began now to recover rapidly. Increaſed the ferr. vitr. to gr. xxx. kali myrrh a. ℈ij. tinc. ſcil. ſpt. æth. nit. aa. ℨiijſs. aq. menth. ℥vi. M. This quantity was daily perſevered in till the 13th of July, on which day he quitted the Hoſpital perfectly reſtored to health, all the ſymptoms having gradually diſappeared; even the cough and expectoration appeared wholly removed. There was a variety of ſmall occurrences reſpecting this and the preceding patient, which I have omitted, to avoid prolixity; but each had bliſters applied to the ſcrob. cord.

From the fortunate termination of theſe bad caſes, are we not juſtly entitled to conclude, that the above plan of cure will commonly prove ſucceſsful for the removal of Dropſy as a general diſeaſe, and even when combined with incipient phthiſis, or a morbid ſtate of the lungs? But where it depends on long eſtabliſhed ſcirrhus of the liver, or other viſcera, on polypi of the heart and great blood veſſels, on compreſſion from fixed and indolent tumours, or, in ſhort, when it is ſymptomatic only of ſome internal incurable malady; then, indeed, the probability of ſucceſs will be leſs apparent. I am,

Gentlemen,

Your obedient humble ſervant,

*July* 20, 1800. JAMES MAGENNIS, M.D.

Phyſician to the Royal Hoſpital, Plymouth.

